# Linking Nonalcoholic Fatty Liver Disease and Brain Disease: Focusing on Bile Acid Signaling

**DOI:** 10.3390/ijms232113045

**Published:** 2022-10-27

**Authors:** Zi-Lin Ren, Chang-Xiang Li, Chong-Yang Ma, Dan Chen, Jia-Hui Chen, Wen-Xiu Xu, Cong-Ai Chen, Fa-Feng Cheng, Xue-Qian Wang

**Affiliations:** 1 School of Traditional Chinese Medicine, Beijing University of Chinese Medicine, Beijing 100029, China; 2 School of Traditional Chinese Medicine, Capital Medical University, Beijing 100069, China; 3 Dongzhimen Hospital, Beijing University of Traditional Chinese Medicine, Beijing 100700, China

**Keywords:** NAFLD, brain disease, bile acids, FXR, GPBAR1

## Abstract

A metabolic illness known as non-alcoholic fatty liver disease (NAFLD), affects more than one-quarter of the world’s population. Bile acids (BAs), as detergents involved in lipid digestion, show an abnormal metabolism in patients with NAFLD. However, BAs can affect other organs as well, such as the brain, where it has a neuroprotective effect. According to a series of studies, brain disorders may be extrahepatic manifestations of NAFLD, such as depression, changes to the cerebrovascular system, and worsening cognitive ability. Consequently, we propose that NAFLD affects the development of brain disease, through the bile acid signaling pathway. Through direct or indirect channels, BAs can send messages to the brain. Some BAs may operate directly on the central Farnesoid X receptor (FXR) and the G protein bile acid-activated receptor 1 (GPBAR1) by overcoming the blood–brain barrier (BBB). Furthermore, glucagon-like peptide-1 (GLP-1) and the fibroblast growth factor (FGF) 19 are released from the intestine FXR and GPBAR1 receptors, upon activation, both of which send signals to the brain. Inflammatory, systemic metabolic disorders in the liver and brain are regulated by the bile acid-activated receptors FXR and GPBAR1, which are potential therapeutic targets. From a bile acid viewpoint, we examine the bile acid signaling changes in NAFLD and brain disease. We also recommend the development of dual GPBAR1/FXR ligands to reduce side effects and manage NAFLD and brain disease efficiently.

## 1. Introduction

One-quarter of the world’s population is now affected by NAFLD, the most widespread cause of chronic liver disease [1], which is known to be intimately related to the metabolic syndrome’s elements [2]. A more serious stage of NAFLD, non-alcoholic steatohepatitis (NASH), can lead to advanced liver disease, cirrhosis, and hepatocellular carcinoma [3]. The primary driver of NAFLD is overnutrition, and the pathogenic pathways of NAFLD are influenced by multiple metabolic, genetic, and microbiome-related factors [4]. Although the main long-term effect of the metabolic syndrome is thought to be cardiovascular disease, other organs, including the brain, may also be affected [5]. In addition to an elevated risk of stroke [6], NAFLD patients may also have modest or early cognitive impairment [7]. NAFLD is also an independent risk factor for depression [8], which affects patients’ quality of life [9].

As multipurpose signaling molecules, BAs have the capacity to control lipid, glucose, and energy metabolism [10,11], and their aberrant metabolism is a significant contributor to NAFLD. It is possible that bile acids can govern the brain because bile acid receptors are expressed in the brain as well [12,13]. As a result, we suggest that NAFLD influences the bile acid signaling system, which in turn influences the emergence of brain illness. The importance of bile acids in neurological illnesses has been mentioned in a number of recent reviews, but they have not explored, in detail, how the bile acid signaling pathways connect NAFLD and brain problems [14,15,16]. We highlight brain disease in this review as an extrahepatic manifestation of NAFLD, linking the two conditions via the bile acid signaling system.

## 2. Bile Acid of Liver Damage in NAFLD

**Figure 1:**
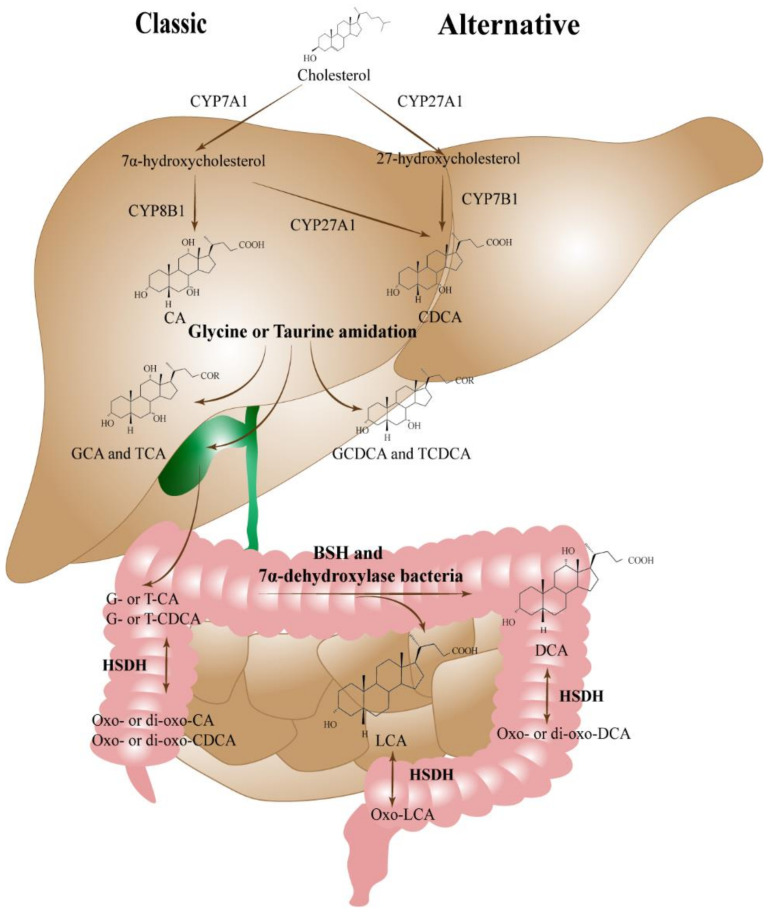
Bile acid biosynthesis.

In hepatocytes, the neutral/classic pathway is largely used to make BAs, whereas the acidic/alternative pathway is used in a smaller number of cases. Cholesterol 7a-hydroxylase (CYP7A1) is mostly rate-limiting and controls the classical route. The alternative method uses cytochrome P450 27A1 (CYP27A1) to transform cholesterol into (25R)-26-hydroxycholesterol [17]. The major Bas: cholic acid (CA) and chenodeoxycholic acid (CDCA), are produced via these two routes [14,15]. An increased bile acid ionization, amphipathic characteristics, and solubility, which are transported to and stored in the gallbladder, are produced when Bas, such as CA and CDCA are conjugated to glycine and taurine [18,19]. Cholecystokinin (CCK), a hormone secreted by the pancreas after eating, causes the gallbladder to contract and release BAs into the digestive system [18]. About 95% of bile acid (BA) molecules are reabsorbed in the terminal ileum and enter the liver via the portal vein, creating a pool of circulating BAs [20]. The intestinal flora’s action causes the remaining BAs to enter the colon and produce the primary secondary Bas: deoxycholic acid (DCA) and lithocholic acid (LCA) [19,20]. Other bile acids, including ursodeoxycholic acid (UDCA), can be formed through additional chemical changes [21]. Figure 1 summarizes the biosynthesis of bile acids.

Patients with NAFLD/NASH have been found to have altered total BA levels, as well as composition. Adults with liver biopsy-confirmed NAFLD and NASH had their BA levels tested, and these levels were dose-dependently linked with the histological characteristics of NAFLD/NASH [22,23]. As the stage of liver inflammation and fibrosis increased, the proportion of primary bound BAs increased [22,23], especially the primary BAs bound to glycine [22]. It has been shown that patients with NASH have elevated levels of BAs, mainly hydrophobic and more cytotoxic secondary BAs [24,25]. The ratio of conjugated BAs changed together with the absolute concentration of the BAs. In early chronic liver disease (non-alcoholic fatty liver [NAFL] + NASH), one investigation verified that levels of glycocholic acid (GCA)/taurocholic acid (TCA) and glycodeoxycholic acid (GDCA)/taurodeoxycholic acid (TDCA), changed with the severity of the disease [26]. NAFLD resulted in a significant elevation of certain circulating BAs, which appeared to be based on an activation of the oxysterol 7-α hydroxylase (CYP7B1)-dependent alternative BA synthesis pathway [27], and the downregulation of the levels of uptake and export transporters of the BAs in the liver [28]. Additional genomic studies have connected higher circulating BAs in NAFLD to the genetic variation in genes [22]. Furthermore, there are some potential medicinal uses for bile acids. In preclinical research, specific gene clusters regulating drug metabolism, lipid homeostasis, and BA were strongly influenced by CDCA [29]. In the meantime, the leptin-deficient obese mice with UDCA may control the hepatic energy balance and white adipose tissue macrophage polarization [30], as well as restore the gut microbiota and reduce liver inflammation in a non-alcoholic steatohepatitic mouse model [31]. The ability of UDCA to lower alanine aminotransferase (ALT) in NAFLD was demonstrated by a meta-analysis of randomized clinical studies [32]. Additionally, after six months of treatment, UDCA reduces women’s 10-year atherosclerotic cardiovascular disease (ASCVD) risk and carotid intima-media thickness (CIMT), in the overall sample [33].

The two metabolic processes, known as the classical/neutral and alternative/acidic pathways, are used in the liver to synthesis CA and CDCA from cholesterol. Following their secretion into the bile ducts, these two primary BAs are then transferred to the gut where the intestinal microbiota transforms them into the secondary bile acids DCA and LCA, or in their corresponding oxo- and dioxygenic derivatives. 

## 3. Bile Acid Alterations and Their Function in Brain Disease

**Figure 2:**
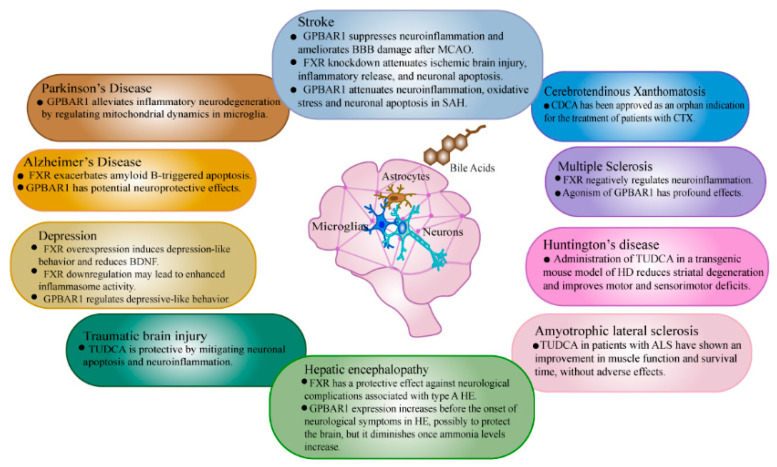
Summary of the effects of bile acid-mediated signaling on brain disease.

Extensive evidence obtained in recent years has revealed that NAFLD could be linked to a lower cognitive performance [34], mood imbalances [35] (in particular, depression and anxiety), cerebrovascular alterations [36], and a low total cerebral volume [37]. When NAFLD develops, a number of pro-inflammatory cytokines and chemokines are released into the bloodstream, starting the coagulation cascade pathway and causing the endothelial dysfunction and cerebral atherosclerotic disease [38,39,40,41]. Overnutrition contributes to BBB damage, the development of neuroinflammation, and finally memory and cognitive impairment [42]. Simultaneously, the neurotransmitter synthesis and signaling are disrupted, further promoting depression-like behavior [43]. Risk factors for NAFLD have been hypothesized to hasten cerebral small artery disease, resulting in white-matter lesions, cerebral microbleeds, and brain shrinkage [5]. BAs, as the main organic solute in bile, play a vital role in the liver and other tissues, which are essential for nutrients in the small intestine and for liver metabolism. We take BAs as an entry point to further link NAFLD and brain disease. Figure 2 summarizes the consequences of bile acid-mediated signaling for brain disorders. 

### 3.1. Stroke

The most common cause of mortality and lasting disability in the world is stroke [44]. The lifetime risk of stroke is about 25% for both men and women worldwide, at age 25, with East Asia, Central Europe, and Eastern Europe having the highest rates [45]. An increasing series of clinical studies have explored the relationship between stroke and NAFLD. The incidence of NAFLD in stroke patients, ranged from 40 to 64 percent in the majority of studies, and hepatic steatosis is typically identified by imaging methods (ultrasound or transient elastography) or liver enzymes [46,47,48,49,50]. Only one study shows a substantially lower prevalence of hepatic steatosis, at about 8 percent [51]. More studies have revealed a link between NAFLD and a higher risk of stroke [6,52,53]; however, it is unclear if this link extends to more severe strokes and worse outcomes [47,48,51]. The variable definitions of NAFLD, the low sensitivity of the tests used to diagnose NAFLD, and the decision of whether to account for potential confounding variables and the number of NAFLD patients, may be the causes of these inconsistent results. Additionally, a study from the REasons for Geographic and Racial Differences in Stroke (REGARDS) that found a link between the baseline fatty liver biomarkers and future stroke in women, but not men, in the United States, also addressed sex differences [53]. Based on the REGARDS study, a different study revealed that advanced liver fibrosis may only be a risk factor for ischemic stroke in women [54]. Additionally, a system analysis revealed that NAFLD was linked to a higher incidence of cerebrovascular accidents (CVAs) in both Caucasians and Asians [55]. These inconsistent results could be the result of race and gender-related hormonal variations.

Primary bile acid levels in rat brain tissue, used in ischemia/reperfusion models, were considerably lower than those in the control group [56]. Two hours after a traumatic brain injury, the bile acid receptors and transporters were downregulated and the hepatic acute phase response was activated [57]. Young patients with stroke had a significantly different bile acid composition from the control group, according to a metabonomics analysis [58]. As a glycine-conjugated bile acid, the levels of GCDCA were significantly elevated in young ischemic stroke patients [58]. A component of bile acid, CA, controls neuroinflammation, oxidative damage, and growth factors, leading to the recovery of the BBB function and neuronal phenotype [59]. Additionally, TUDCA has been demonstrated to have neuroprotective effects in a rodent model of a hemorrhagic brain injury, by preserving the stability of the mitochondrial membranes or inhibiting the mitochondrial disturbance [60], reducing apoptosis [60], inhibiting endoplasmic reticulum stress, and reducing neuronal pyroptosis [61]. Similarly, TUDCA plays a role in ischemic brain injury by improving the mitochondrial function and reducing apoptosis [62]. Additionally, through negatively affecting the nuclear factor (erythroid-derived 2)-like 2 (Nrf2) signaling pathway, TUDCA may reduce oxidative stress, the inflammatory response, and apoptosis in rats with an acute cerebral infarction [63]. TUDCA has been shown to diminish the expression of chemoattractants and vascular adhesion proteins, as well as the activation of glial cells and the ability of microglia to migrate [64]. NAFLD has been linked in clinical studies to an increased risk of stroke, and preclinical research has demonstrated the neuroprotective properties of several bile acids, including CA and TUDCA.

### 3.2. Depression

Depressive disorders, also referred to as mood disorders or anhedonia, are common and debilitating mental health conditions [65], which account for the majority of years spent in a disabled state, globally [66]. There is growing evidence that suggests a connection between depression and the existence and even severity of NAFLD [67,68,69,70]. NAFLD is also regarded as a standalone depression risk factor [8,71]. A meta-analysis from 2020 showed that the co-prevalence of depression was 18.21% in NAFLD patients and 40.68% in NASH patients [68]. Meanwhile, numerous investigations conducted in the community, revealed that people with depression had a twofold increased risk of a metabolic syndrome, compared to those without a history of melancholy [72,73]. Depression was linked to a more severe hepatocyte ballooning in NAFLD patients, and there was a dose-dependent relationship between the intensity of depressed symptoms and the grade of the hepatocyte ballooning [74].

Over-activation of the intestinal signaling pathways FXR receptor-FGF15 and hepatic apoptosis signal-regulating kinase1 (ASK1) in dextran sulphate sodium-induced depressive rats, suggests hepatic metabolic disturbances [75]. The chronic unpredictable mild stress (CUMS) model group had considerably higher serum bile acid levels [76]. Liver metabolomics studies showed that TUDCA increased [77,78], while TDCA and TCDCA decreased in the CUMS group [79]. The fecal metabolomics investigation revealed that stress significantly reduced the levels of CA, DCA, and CDCA [80]. The antidepressant mechanism may be connected to the reduction of neuroinflammation [81,82], oxidative/nitrosative stress [81,82], and endoplasmic reticulum stress [82] in the brain, and TUDCA proved successful in alleviating depression-like behavior in rats. In an experiment verifying that interferon alpha (IFN-α) causing depression, via the modulation of glucocorticoid and serotonin receptors, the effect of IFN-α on the glucocorticoid receptors was abolished when used in combination with TUDCA [83]. Interestingly, TUDCA did not have any effect on the expression level of the serotonin receptor 1A [83]. People who suffer from depression are more likely to develop a metabolic syndrome, and TUDCA, one of the bile acids, has been demonstrated, in animal experiments, to reduce depression-like behavior.

### 3.3. Alzheimer’s Disease

The most prevalent form of late-life dementia, Alzheimer’s disease (AD), is a neurodegenerative condition with etiologies related to the accumulation of beta-amyloid plaques and neurofibrillary tangles, synapse and neuronal loss, and cerebral shrinkage [84]. In comparison to the control group, both APP/PS1 and *APP^NL-G-F^* animals have significantly different amounts of conjugated and unconjugated primary bile acids, indicating that the liver’s overall metabolic balance is disturbed [85]. Hepatic metabolism has been discovered as a biological factor in the neuropathological resilience in Alzheimer’s disease by a broad genetic investigation of resilience [86]. Moreover, NAFLD might promote the pathological AD indications in an AD model and trigger AD signs in wild-type mice [87]. When compared to the control group, serum and brain tissue from AD patients had higher ratios of secondary to primary bile acids, such as DCA:CA or GCDCA:CA [88,89,90]. Surprisingly, LCA grew 3.2-fold within 8–9 years, while being converted to AD as a secondary bile acid [91]. Additionally, it was shown that the amount of the bile acid precursor 7α,25-dihydroxy-3-oxocholest-4-en-26-oic acid was reduced in the cerebrospinal fluid (CSF) of AD patients [92]. Lower serum levels of primary BAs and 7α-hydroxycholesterol (7α-OHC) were associated with a faster buildup of white-matter lesions, a faster brain atrophy, and a higher amyloid deposition in the brain, mostly in males [93], possibly as a result of estrogen’s protective effects [94]. In fibroblasts, UDCA has been found to reduce the number of long mitochondria and boost the mitochondrial membrane potential and respiration [95]. Additionally, when activated by β-amyloid peptide (Aβ), UDCA may block the production of the nuclear factor-κB (NF-κB)-dependent genes in microglia [96]. For the treatment of AD in mice, TUDCA, an endogenous bile acid created by the conjugation of UDCA with taurine, has demonstrated a significant therapeutic potential. TUDCA can improve the cognitive function in mice by inhibiting apoptosis [97,98], improving neuroinflammation [99], preventing the reduction in dendritic spine numbers [99], and enhancing glucose homeostasis [100]. Additionally, rats treated with aluminum chloride (AlCl_3_) have an improved hippocampal insulin sensitivity due to CDCA [101]. In conclusion, some bile acids have also been demonstrated to be useful in preclinical trials of AD, including UDCA, TUDCA, and CDCA, and liver metabolism plays a significant role in AD patients. 

### 3.4. Parkinson’s Disease

Parkinson’s disease (PD) is a long-term, progressive neurodegenerative condition that manifests in both the motor and non-motor systems [102]. Patients with PD typically present with resting tremor, rigidity, bradykinesia, and stooping posture [102]. Lewy bodies and a loss of dopaminergic neurons in the substantia nigra are symptoms of PD [103]. Chronic inflammation outside of the brain, such as NAFLD, was sufficient to induce neurodegeneration without genetic predisposition [87]. While this is happening, BAs are a significant aberrant biochemical route in PD patients, whether they are seen in the plasma, cerebral fluid, or intestinal tissues [104,105,106,107,108,109]. PD patients had significantly elevated plasma CA/CDCA and decreased glycoursodeoxycholic acid (GUDCA) [104,107], and the intensity of motor complaints was linked with taurine-conjugated bile acids [106]. More carefully differentiating the different subtypes of PD patients, GDCA was significantly higher and TCA was significantly lower in leucine-rich repeat kinase 2 (LRRK2) PD patients, GCDCA was significantly lower in sporadic PD (sPD) patients, and TCA was significantly higher in LRRK2 PD compared to sPD patients [109]. However, an increase in unconjugated bile acids (CA, DCA, and LCA) has also been reported in patients with LRRK2 PD [110]. Additionally, an examination of the data from Parkinson’s disease genome-wide association studies using an empirical Bayesian Lasso showed three significant pathways, including the main pathway for bile acid production [111]. 7α,(25R)26-dihydroxycholesterol and a second oxysterol 7α,x,y-trihydroxycholest-4-en-3-one (7α,x,y-triHCO) were found to be considerably higher in PD CSF, suggesting that the acidic pathway of bile acid production has been activated [108]. Unexpectedly, the appendix and ileum of Parkinson’s patients had higher levels of DCA and LCA, and hydrophobic and secondary bile acids [105]. 

Several experiments have shown that TUDCA could prevent the dopaminergic neuronal damage and improve Parkinsonian dyskinesia by inhibiting neuroinflammation [112,113,114], resisting oxidative stress [114,115,116,117], reducing endoplasmic reticulum stress [118], improving mitochondrial dysfunction [113,115], reducing apoptosis [119], and preventing autophagy [114]. UDCA has been validated not only in experimental animal models of prodromal PD [120,121,122] but also in Parkinson’s patients for its pharmacokinetics and safety [123]. UDCA may enhance the mitochondrial activity in PD patients [123], and a phase II, two-center, double-blind, randomized, placebo-controlled trial of UDCA in 30 individuals with early PD, was concluded in July 2021 [124].

The changes in bile acids in brain disease are not exactly similar to those in NAFLD. Patients with NAFLD have an elevated proportion of primary bound bile acids, especially those bound to glycine. Similarly, bile acids bound to glycine, such as GCDCA, are elevated in patients with young ischemic strokes. However, GUDCA has decreased in PD patients. In preclinical studies of depression, it was shown that taurine bound bile acids were altered. Additionally, in both serum and brain tissue from AD patients, the ratio of secondary to primary bile acids increased. UDCA has a therapeutic effect on NAFLD and has a positive effect on the risk of ASCVD. Additionally, UDCA has been shown to be safe in PD patients and is crucial in the treatment of neurodegenerative illnesses. More clinical trials are required to confirm TUDCA’s efficacy; however, it has demonstrated considerable therapeutic potential for the treatment of cerebrovascular diseases, neurodegenerative diseases, and various mental diseases. 

## 4. Bile Acid Signaling to the Central Nervous System

Among the class of nuclear and cell-surface receptors, known as bile acid-activated receptors (BARs), FXR and GPBAR1, are the most distinctive. The expression of BARs is most common in hepatocytes and ileum, as well as in the brain [12,13,125], implying that the ligands could directly bind to the receptor after crossing the BBB. Furthermore, the intestinal FXR and GPBAR1 activation causes the release of FGF19 and GLP-1, both of which communicate with the brain [126]. The role of additional receptors that might bind bile acids and are located in the brain, is summarized in Table 1.

### 4.1. Sending Bile Acid Signaling to the Central Nervous System via a Direct Pathway

BAs can reportedly pass the BBB, both conjugated and unconjugated [125,127,128]. The lipid layer of the BBB can be damaged by BAs at high concentrations (≥1.5 mM), while at low concentrations (0.2–1.5 mM), CDCA and DCA can damage the tight junction protein through the Rac1-dependent processes [129,130]. Additionally, the BA uptake by neurons is facilitated by bile acid transporters produced in the central nervous system, such as the apical sodium-dependent bile acid transporter (ASBT) [57,131]. Meanwhile, bile acid synthesis-related enzymes and intermediates have been observed, indicating that potentially a portion of the bile acid biosynthetic pathway occurs in the brain [132,133,134]. Numerous investigations have demonstrated that bile acid receptors are dispersed throughout the brain, for instance, GPBAR1 is expressed in cortical neurons, astrocytes, and microglia in the brain [135,136], and FXR is expressed in prefrontal cortical neurons and hippocampal neurons [137,138]. Thus, bile acid ligands, that have the ability to cross the BBB can bind directly to the receptor. Although their primary function is still the removal of cholesterol, BAs also have a range of effects on the physiology of the brain, neurogenesis, neurotransmission, and neuroendocrine responses in addition to acting as neurotrophic agents [14,16].

**Table 1:** Bile acid receptors found in the brain.

Receptor	Cellular Localization	Bile Acid Ligands	Main Function	References
FXR	Prefrontal cortical neurons, hippocampal neurons	CDCA, DCA, LCA, CA	The function of FXR in the brain is still paradoxical. FXR knockdown attenuated neuronal apoptosis in ischemic brain injury. FXR aggravated amyloid-β-triggered apoptosis by modulating the CREB-BDNF pathway in vitro. By causing CRTC2 to translocate into the cytoplasm and disrupt the CREB-BDNF signaling pathway in the hippocampus nucleus, FXR played a role in the pathophysiology of depression. In rats, the hippocampal BDNF expression is decreased and depression-like behavior caused by CUMS is reversed by the hippocampus FXR knockdown. FXR signaling pathway was neuroprotective in mice with depression. In the hippocampi of AlCl3-treated rats, CDCA was successful in enhancing insulin sensitivity.	[101,137,138,139,140,141]
GPBAR1	Neurons, astrocytes, microglia	LCA, DCA, CDCA, CA	In a rodent model of subarachnoid hemorrhage, neuroinflammation, oxidative stress, and apoptosis, as well as BBB damage and neuroinflammation after MCAO, were all reduced by the GPBAR1 activation. The locomotor activity of mice was increased and obesity, depression, and cognitive decline were all protected by the olive leaf extract, which contains a GPBAR1 agonist. Through the hippocampal CA3 pyramidal neurons afferent to the dorsolateral septum, GPBAR1 altered depressive-like behaviors. GPBAR1 activation alleviated inflammatory neurodegeneration in a mouse model of PD by regulating the mitochondrial dynamics in microglia.	[135,136,142,143,144,145,146,147]
PXR	Brain endothelial cells, hippocampal neurons	LCA	Nonylphenol had neurotoxic and apoptotic effects on mouse hippocampus cells, and PXR was involved in the spread of those effects. Knockdown expression of PXR in the midbrain of Long–Evans rats lead to impaired mating behavior and reduced hippocampal BDNF levels.	[148,149]
VDR	Neurons, glia	LCA	In rat primary astrocytes, the VDR activation controlled the quantities of glutathione and gamma-glutamyl transpeptidase produced. VDR activation suppressed the inducible synthesis of nitric oxide and decreased the generation of pro-inflammatory cytokines by the activated microglia. The pathophysiological process of depressive-like behaviors brought on by persistent stress may involve the 25(OH) D and VDR. By increasing the hippocampus BDNF expression, the VDR signaling helped mice with post-stroke depressive symptoms. In a rat model of traumatic brain damage, the VDR activation altered the NADPH oxidase 2 activity and prevented neurological impairments and apoptosis. Brain endothelial P-glycoprotein levels were reduced in PD via a VDR-dependent pathway.	[150,151,152,153,154,155]
S1P2R	Cortical neurons, microglia, astrocytes, hippocampal pyramidal cells, retinal ganglion cells	GCA, GDCA TCA TDCA, TUDCA	S1P2R antagonists improved the neural progenitor cell migration near the brain infarction and reduced the hepatic encephalopathy-related neurological impairment. By weakening the adherens junctions, S1P2R may control BBB permeability.	[156,157,158,159]
α5β1 integrin	Cortical neurons, brain endothelial cells	TUDCA, nor UDCA (UDCA homolog)	Activation of integrin α5β1 promoted angiogenesis in brain endothelial cells under cerebral hypoxia, as well as the vascular endothelial growth factor secretion in MCAO rats. α5β1 integrin influenced the BBB permeability following an ischemic stroke.	[160,161,162]
GR	Neurons, microglia, cortical neurons	GCDCA TCA, TUDCA, UDCA	Inhibiting NF-κB in a glucocorticoid-dependent way throughout the middle stage of depressive-like behavior, GR was beneficial. Both too little and too much GR-mediated signaling hampered the neuronal migration. In neuron-like cells, GR suppressed the production of the brain-derived neurotrophic factor. GR-signaling in ginseng had an anti-inflammatory protective effect on neurodegenerative models.	[163,164,165,166]

Abbreviations: CREB, cyclic AMP (cAMP)-response element-binding protein; CRTC, CREB-regulated transcription coactivator; PXR, pregnane X receptor; VDR, vitamin D receptor; NADPH, nicotinamide adenine dinucleotide phosphate; S1P2R, sphingosine 1-phosphate receptor 2; GR, glucocorticoid receptor.

### 4.2. Sending Bile Acid Signaling to the Central Nervous System via an Indirect Pathway

#### 4.2.1. FXR Signaling

Inhibiting bile acid synthesis in the liver, controlling bile acid circulation between the liver and the intestines, and maintaining a consistent level of bile acids in the body are all possible effects of FXR. Small heterodimer partner (SHP) levels rise and sterol regulatory element binding protein (SREBP)-1c expression decreases as the FXR activation reduces lipid levels [167]. The expression of the human peroxisome proliferator-activated receptor α gene is also encouraged by the FXR activation [168]. In contrast, it is still unknown how FXR affects cholesterol and lipoprotein production in clinical trials, and giving individuals with NASH an FXR agonist raises their cholesterol levels and negatively affects their lipoprotein levels [169]. The transcriptional activity of the protein that binds to the carbohydrate-response element-binding protein (ChREBP), controls the glucose metabolism when FXR is activated [170]. Additionally, in response to the FXR activation, the intestine secretes FGF19, the human homolog of mouse FGF15, which binds to the hepatocytes via the enterohepatic circulation and stimulates the glycogen production [171]. It is known that FGF15/19 inhibits the CREB-peroxisome proliferator-activated receptor γ coactivator-1α (PGC-1α) pathway, which in turn reduces the production of the hepatic gluconeogenic genes [172]. By decreasing the CYP7A1 gene transcription through a jnk-dependent mechanism, FGF15/19 regulates the bile acid homeostasis in the liver by binding to the fibroblast growth factor receptor (FGFR) 4 [173,174].

In the absence of a heparin-binding domain linking it to cells, FGF15/19 could enter the circulation, cross the BBB and act in the brain, but with lower penetration rates, compared with FGF21 [175,176]. Similar results from in vitro investigations showed that middle cerebral artery occlusion therapy dramatically decreased the number of FGF19-positive cells in the cerebral cortex, compared to the sham group [177]. FGFR (including FGFR1c, FGFR2c, and FGFR3c) and the plasma-membrane bound β-klotho co-receptor are both necessary for FGF19 to bind to them [178,179,180]. Strikingly, FGFR4 is unique in that it binds to FGF19 in the presence or absence of β-klotho [181,182]. In contrast to FGFR4, which is only found in the medial habenula, sub-commissural organ, and lateral habenula of rodents, FGFR1–3 are strongly expressed throughout the entire brain [183]. The brain expresses FGFR broadly and abundantly, whereas the β-klotho expression is rather modest and discrete in distribution [183,184,185,186,187]. Along with the forebrain areas, such as the hypothalamus, hippocampus, or amygdala [176], β-klotho is also found in the midbrain areas, including the ventral tegmental area, medial vestibular nucleus, and medial trigeminal neurons [183,184], as well as in the area postrema and nucleus of the solitary tract [183]. The limited co-expression of FGFR1 and β-klotho has been found in cells of the hippocampus CA1-CA3 transition zone, the primary sensory nucleus of the trigeminal nerve, medial trigeminal neurons, and suprachiasmatic nucleus [183]. To our knowledge, however, no research has looked at the co-expression of FGFR2, FGFR3, and β-klotho. As a result, following the activation of the FGFR-β-klotho complex, FGF15/19 may be able to target specific brain regions and cause a signaling cascade response.

#### 4.2.2. GPBAR1 Signaling

The membrane-bound G-protein-coupled bile acid receptor GPBAR1, also known as the Takeda G-protein-coupled bile acid receptor 5 (TGR5), is activated by bile acids in the following order: LCA > DCA > CDCA > CA [188]. Several non-parenchymal cells, such as the Kupffer cells and liver sinusoidal cells (LSC), express GPBAR1 in the liver [189]. In wild-type mice, GPBAR1 inhibits NF-κB, which negatively affects the hepatic inflammatory response [190]. Through a TGR5-cAMP-dependent mechanism, BAs prevent Kupffer cells from producing cytokines, in response to lipopolysaccharide (LPS) [191]. In the bowel, the GPBAR1 activation increases the colonic motility and secretion, decreases the gastric emptying, and encourages the production of GLP-1 from L cells, which in turn encourages the release of insulin from pancreatic β-cells [189,192]. In vascular endothelial cells, GPBAR1 is capable of vasodilatation through the release of nitric oxide (NO) and hydrogen sulfide (H2S), thereby reducing the vascular inflammation and decreasing the development of atherogenesis [192]. Additionally, BAs stimulate GPBAR1 in adipose and muscular tissue, boosting thermogenesis and energy expenditure [193]. 

In addition to direct GPBAR1 ligand binding, endogenous neurosteroids in the brain may also activate GPBAR1 [12]. Furthermore, the bile acid activation of GPBAR1 in the intestine, results in the release of the gut hormone GLP-1, which communicates bile acid signals to many body systems, such as the central nervous system [194,195]. By directly stimulating the GPBAR1 receptors on the basolateral side of the L cells, bile acids may change how much GLP-1 is released [195]. Dipeptidyl peptidase-IV (DPP-IV) breaks down GLP-1 so that only 1/4 of the released amount enters the portal circulation after it diffuses into the lamina propria and is absorbed by a capillary [196]. Another 50–60% is retained in the liver, leaving 10–15% to enter the systemic circulation [197]. DDP-IV is also present in the plasma, and much less GLP-1 may reach the brain via the endocrine pathway [196]. GLP-1 is able to access the brain by simple diffusion [198]. The GLP-1 receptor is widely present in the hypothalamic region of rodents and primates, particularly in the paraventricular nucleus, dorsomedial hypothalamus, and arcuate nucleus [199]. Additionally, the intestinal GLP-1 can communicate with the brain and central nervous system by activating vagal afferent fibers. Afferent sensory nerve fibers coming from the nodose ganglion, which provides impulses to the nucleus of the solitary tract and ultimately to the hypothalamus, may interact with GLP-1 before it enters the capillaries to be destroyed by DPP-IV [200]. The hepatic vagus has been shown to specifically recognize the GLP-1 expressions in the hepatoportal area [201]. Additionally, GLP-1 may communicate with the central nervous system through spinal and local sensory nerves [194]. The fact that GLP-1 is produced by preproglucagon neurons in the lower brainstem, is a key factor [202]. The difficulty of assessing the peripheral GLP-1 that is entering the central nervous system for action increases as GLP-1 is released from the brain.

## 5. Bile Acid Signaling in Brain Disease

### 5.1. FXR Signaling

The FXR expression was elevated in the nuclei of neurons, following cerebral ischemia, but not in microglia, astrocytes, or endothelial cells [139]. When Aβ-triggers neuronal death in differentiated SH-SY5Y cells and mice hippocampus neurons, the FXR expression is increased [137]. Proteomic and metabolomics studies of plasma and hypothalamus in depressed mice, indicated significant changes in the FXR/retinoid X receptor (RXR) activation [203,204]. Chen et al. [141] reported that CUMS dramatically increased the expression of the hippocampal FXR, a key player in the pathophysiology of depression. Furthermore, Hu et al. [140] observed that chronic unexpected stress worsens depressive-like behavior by overexpressing FXR in the hippocampal CA1, but not in the dentate gyrus (DG) or medial prefrontal cortex (mPFC). However, chronic social defeat stress (CSDS)-induced mice had a considerably less FXR expression in the prefrontal cortex (PFC), which increased the inflammasome activity, impaired the neuronal synaptic function, and elevated the caspase-1 activity [138]. Taken together, in disease states, the FXR expression in the brain is region-specific.

In several experimental disease models, the activation and inhibition of the FXR signaling could have counterintuitive or even opposing consequences. In mice, after brain ischemia, the FXR deletion might lower the calcium influx, encourage the neurobehavioral recovery, lessen the ischemic brain injury, reduce the inflammatory release, and attenuate neuronal death [139]. In differentiated SH-SY5Y cells, the FXR overexpression worsened the Aβ-triggered neuronal death, and the FXR agonist 6ECDCA treatment further improved this result [137]. Through a signaling cascade, including CREB/BDNF, FXR controls the Aβ-induced neuronal death in vitro [137]. Chen et al. [141] showed that the overexpression of the hippocampal FXR could reduce the expression of BDNF in naïve rats, Hu et al.’s [140] study found that the FXR–CREB interaction and the CRTC2 cytoplasmic translocation in the CA1 region were essential for depression, which was confirmed by the use of the hippocampus-specific FXR-null mice. However, the antidepressant effect of ganoderic acid A was mediated by the direct inhibition of NOD-, LRR- and pyrin domain-containing 3 (NLRP3) inflammasome activity and the restoration of the FXR expression in the PFC to activate the AMPA receptor phosphorylation and expression, which was completely abolished in mice injected with the FXR-specific inhibitor z-guggulsterone or in FXR-null mice [138]. A powerful FXR activator called CDCA may increase insulin sensitivity in the hippocampi of an AD rat model [101]. According to one study, the enhanced insulin sensitivity was achieved by the CDCA agonistic action to FXR, which was thought to be mediated via the FXR binding to the GLUT4-FXR response element [205]. When considered collectively, the downstream pathways regulated by FXR are diverse and tissue/cell specific, making it more scientific to use the tissue-specific FXR knockdown to validate and prevent altering the expression of FXR in other tissues. FXR is typically known as a ligand-activated nuclear receptor. Nevertheless, it has been demonstrated that FXR also has physical binding functions in the cytoplasm that are unrelated to its usual transcriptional activity [206]. These may be the reasons for the conflicting or even diametrically opposed outcomes that FXR signaling activation and inhibition may have. To fully comprehend the significance of the FXR expression in brain illnesses, more research on the FXR ligand-dependent and ligand-independent action in tissue/cell-specific FXR-null mice is required.

### 5.2. GPBAR1 Signaling

Preclinical studies of the middle cerebral artery occlusion and SAH have shown that the endogenous GPBAR1 expression is elevated in each case [135,142,143,144]. Intriguingly, the hippocampal CA3 pyramidal neurons’ GPBAR1 expression was reduced in the chronic restraint stress (CRS) and CSDS models of depression [145]. There was no discernible difference in the GPBAR1 expression between the mouse model of the 1-Methyl-4-phenyl-1,2,3,6-tetrahydropyridine-induced motor impairments and cognitive impairment and the control group [146]. However, the GPBAR1 expression is significantly decreased in mice with an Aβ1-42-induced cognitive impairment [207]. Although the expression of GPBAR1 was inconsistent in the above diseases, INT777, a specific agonist of GPBAR1, has been shown to have a partial alleviation in some brain diseases. Through the Pellino3 suppression of caspase-8/NLRP3, INT777 dramatically reduced brain damage and enhanced the neurobehavioral outcomes following MCAO [142]. Inhibition of GPBAR1 or Pellino3 rendered INT777’s anti-inflammatory actions ineffective [142]. Activating GPBAR1 could reduce damage to the BBB and improve the neural function through the BRCA1/sirtuin-1 (Sirt1) signaling pathway [143]. Additionally, via the TGR5/cAMP/PKA signaling pathway, the INT777 activation of GPBAR1 could decrease the activation of the NLRP3-apoptosis-associated speck-like protein containing a CARD (ASC) inflammasome in microglia, reduce brain edema, and enhance the short-term neurobehavioral function following SAH [144]. Similarly, INT777 reduced oxidative stress and neuronal death after SAH via the cAMP/PKCε/aldehyde dehydrogenase 2 (ALDH2) signaling pathway, which was blocked by the GPBAR1 and ALDH2 knockdown [135]. Alternately, INT777 dramatically decreased apoptosis, enhanced the synaptic dysfunction, and ameliorated the cognitive impairment brought on by Aβ1-42 [207]. By reducing the mitochondrial damage and autophagic dysfunction in microglia, INT777 reduced the tumor necrosis factor α (TNFα) release and relieved the PD neurodegeneration [146]. In addition, the genetic overexpression of GPBAR1 or the re-expression of GPBAR1 in the CA3 pyramidal neurons, and the intra-CA3 infusion of INT-777, all significantly improved the depression-like behavior of mice via the CA3 pyramidal neurons→somatostatin-GABAergic neurons of the dorsolateral septum transmission [145]. In addition to the synthetic agonists of GPBAR1, some natural agonists of GPBAR1 could also improve the cognitive function and alleviate depression. Working memory losses after consuming a high-fat diet and being inactive were avoided by using olive leaf extract, which contains oleanolic acid, a powerful GPBAR1 agonist [147]. TUDCA, a taurine conjugate of UDCA, together with GPBAR1/TGR5-boosted cAMP levels in microglia that triggered anti-inflammatory markers while reducing the pro-inflammatory ones [208]. It has been reported that TUDCA reduces neuronal apoptosis and improves the neurological function after SAH through the TGR5/sirtuin-3 (SIRT3) signaling pathway, and TGR5 small interfering RNA (siRNA), could eliminate the protective effect of TUDCA [209]. Whether this could explain the neuroprotective effect of TUDCA in animal models of several brain diseases mentioned above, needs further investigation.

## 6. Therapeutic Targeting of the Bile Acid Signaling

### 6.1. FXR Agonists

A number of FXR agonists have been developed and tested in clinical trials for the treatment of NASH/NAFLD, in light of the efficacy of the FXR ligands, as a therapy method. Synthetic agonists in clinical trials are summarized in Table 2. The strongest endogenous FXR agonist in humans is CDCA, which is also followed by DCA, LCA, and CA [210].

In a 72-week phase IIb experiment, obeticholic acid (OCA) was found to improve the histological characteristics of the NASH liver (such as hepatocyte ballooning, steatosis, and lobular inflammation) (NCT01265498). The homeostasis model assessment of insulin resistance (HOMA-IR) and insulin alterations were also significantly worsened by the OCA administration, along with dyslipidemia and pruritus. However, the lipoprotein levels improve after the drug discontinuation [169]. In a randomized global phase III study, OCA 25 mg significantly improved the NASH fibrosis levels, but the incidence of pruritus could reach more than half (NCT02548351) [211]. In pruritus, a phase I study of the interaction between Linerixibat and OCA, is being studied in healthy adults (NCT05133830). Additionally, for dyslipidemia, data from a small-scale phase II investigation showed that atorvastatin (10 mg/day) treatment in NASH patients following four weeks of OCA, led to LDL-C levels below the baseline (NCT02633956).

Clinical trials are currently favoring Cilofexor, a strong non-steroidal FXR agonist. In NASH patients, hepatic steatosis was dramatically reduced by 30 mg and 100 mg of Cilofexor; however, liver fibrosis was not significantly improved. In total, 14 percent of individuals on Cilofexor 100 mg, experienced moderate-to-severe pruritus, compared to 4 percent of those taking a placebo (NCT02854605). Additionally, Cilofixol was introduced in a combination strategy with Selonsortib and Firsocostat, in a completed phase II trial. It has been demonstrated that Cilofexor and Firsocostat together were well tolerated, improved NASH activity, and may have an antifibrotic effect (NCT03449446).

Tropifexor has been shown to be safe in healthy human subjects in a phase I trial, and in phase II trials in adult patients with NASH and hepatic fibrosis, the effectiveness of the combination of Tropifexor and Licogliflozin (NCT04065841) and Tropifexor and Cenicriviroc (NCT03517540) is being investigated. EDP305 was shown to be able to lower ALT serum levels and liver fat content in a randomized placebo-controlled experiment. Pruritus occurred in 50.9% and 9.1% of patients in the 2.5 mg and 1 mg groups (NCT03421431), respectively. Other FXR agonists now being tested in phase II clinical studies include Px-104, EYP001, MET409, and TERN-101 (Table 2). For the treatment of brain disorders, the FXR agonists are currently in the preclinical stages of research; their effectiveness in humans is not yet known.

### 6.2. FXR Antagonists

Natural FXR antagonists include muricholic acids (α-β-MCA) [212], glycine-β-MCA [213], guggulsterone [214], stigmasterol [215], and marine steroids [216]. Intestinal FXR antagonists have been shown in numerous studies to decrease the ceramide release to lessen the hepatic triglyceride buildup, and they are being investigated in preclinical disease models, as possible therapeutics for metabolic illnesses [217,218]. In brain disease, the knockdown of FXR or the silencing of the FXR expression in specific regions, improves symptoms and alleviates inflammation and neuronal apoptosis [139,140,141]. Notably, it is unclear if intestine FXR antagonists have a positive impact on brain illness and whether these modifications to the BA composition have negative effects, following the long-term FXR suppression.

### 6.3. GPBAR1 Agonists

GPBAR1 receptors are activated by bile acids and by natural substances, such as oleanolic acid, ursolic acid, and betulinic acid. Preclinical research has suggested that INT777 may be helpful in the treatment of NAFLD or other brain disorders. In a mouse model of steatohepatitis, BAR501, a specific GPBAR1 agonist, might repair liver and vascular damage [219]. A phase IIa trial showed that the glucose effects of SB-756050 + sitagliptin, were comparable to those of sitagliptin alone and SB-756050 exhibited nonlinear pharmacokinetics (NCT00733577).

### 6.4. Dual GPBAR1/FXR Ligands

**Figure 3:**
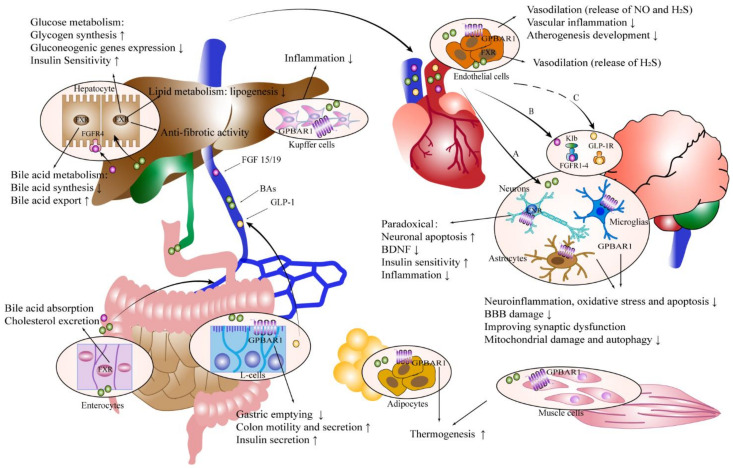
Potential molecular targets of single and dual GPBAR1/FXR agonists in NAFLD and brain illnesses include FXR and GPBAR1. BAs in the liver trigger the activation of FXR, which then has a number of downstream effects, including the prevention of adipogenesis, the decrease in bile acid production, the encouragement of gluconeogenesis, the increase in gluconeogenesis, and anti-fibrosis. Numerous non-parenchymal cells in the liver, including Kupffer cells, express GPBAR1, which may lessen the inflammatory response in the liver. FXR increases bile acid absorption and cholesterol excretion in the terminal ileum. Additionally, FXR promotes the synthesis of FGF15/19, which functions by interacting with FGFR4 in hepatocytes. Additionally, activating GPBAR1 in the intestine increases the colonic motility and secretion, decreases stomach emptying, and stimulates the production of GLP-1 from the L cells, which in turn stimulates the release of insulin from the pancreatic β-cells. In vascular endothelial cells, GPBAR1 is capable of vasodilatation through the release of NO and H2S, reducing the vascular inflammation and decreasing the development of atherogenesis. Furthermore, FXR is capable of vasodilatation through the release of H2S. BAs could use both direct and indirect channels to communicate with the central nervous system (CNS). (A) BAs in the colon bypass the enterohepatic circulation and breach the BBB to reach the systemic circulation where they interact with brain receptors. (B) Only a small portion of FGF19 released by the enterocytes makes it to the portal vein and then into the body’s circulatory system. In order to interact with the brain’s FGF receptors (1–4) and bind β-klotho to create stable complexes, FGF19 must pass the BBB. (C) GPBAR1 activation results in the release of GLP-1 in the L-cells, which DPP-IV swiftly breaks down. The amount of intact GLP-1 that reaches the brain and interacts with the GLP-1 receptor (GLP-1R) is debatable. FXR plays a contradictory role in the brain by boosting insulin sensitivity, lowering inflammation, and decreasing BDNF, while also increasing cell death. In the brain, GPBAR1 reduces neuroinflammation, oxidative stress and apoptosis, decreases BBB injury, improves synaptic dysfunction, and reduces the mitochondrial damage and autophagy. Additionally, BAs stimulate GPBAR1 in adipose and muscular tissue, which raises thermogenesis and energy expenditure.

Two dual GPBAR1/FXR agonists, BAR502 and INT767, both prevent liver injury in preclinical experiments in NAFLD [189] (Figure 3). In contrast to BAR502, which has a modest preference for GPBAR1, INT767 is a preferential FXR agonist [189]. INT767 significantly reduces the atherosclerosis formation in preclinical studies [220,221]. In addition, a phase I study on safety, tolerability, pharmacokinetics, and pharmacodynamics of BAR502 is to be conducted in healthy subjects in 2022 (NCT05203367). The FXR antagonistic/GPBAR1 agonistic compound is in development [222].

## 7. Conclusions and Perspectives

Brain disease may be an extrahepatic manifestation of NAFLD. For NAFLD, the therapy that focuses on hepatic steatosis and fibrosis is simplistic, and the targets of currently developed drugs may be limited. In contrast, anti-inflammatory and anti-fibrotic effects, and the improvement of systemic metabolic disorders may be more consistent with a multi-targeted treatment model for the disease. Additionally, BAs have neuroprotective qualities, and direct and indirect pathways are included in the BA signaling to the brain. The therapeutic implementation of BAs and targeting BA-mediated signaling in brain disease cannot be overemphasized. FXR and GPBAR1 are activated by ligands to regulate not only the liver but also the whole body. However, currently developed agonists of FXR have achieved some efficacy in improving the liver, while affecting the lipid and systemic metabolism. Dual GPBAR1/FXR ligands may be more advantageous than the single FXR or GPBAR1 ligands for systemic metabolism and dyslipidemias. The improvement of dual GPBAR1/FXR ligands for brain disease still needs to be proven in clinical trials. We acknowledge that bile acid signaling may not link all brain diseases and NAFLD, but the better understanding of the BA signaling and its role in the brain will improve our comprehension of this axis, which leads to the development of new therapeutic strategies to manage related disorders.

## Figures and Tables

**Table 2:** FXR synthetic ligands and their clinical status.

Agonist	Clinical Trials	Status
Obeticholic acid (6-ECDCA, INT747)	NCT01265498	Phase IIb: OCA as a FXR ligand in a NASH treatment trial (FLINT)
	NCT03836937	The function of OCA in NAFLD patients with elevated ALT
	NCT02548351	Phase III: Randomized global study to assess the impact of OCA for fibrosis on NASH
	NCT03439254	Phase III: Evaluating the OCA’s safety and effectiveness in patients who have compensated cirrhosis, as a result of NASH
Tropifexor (LJN452)	NCT03681457	Phase I: Tropifexor pharmacokinetics in patients with mild, moderate, and severe hepatic impairment
	NCT04065841	Phase II: Patients with NASH and hepatic fibrosis: efficacy, safety, and tolerability of the combination of Tropifexor and Licogliflozin and each single agent
	NCT03517540	Phase II: Adult treatment for NASH and liver fibrosis using LJN452 and Cenicriviroc: a study of safety, tolerability, and effectiveness
Cilofexor (GS-9674)	NCT02808312	Phase I: Adults’ pharmacokinetics and pharmacodynamics of Cilofexor in those with normal and abnormal liver function
	NCT02654002	Phase I: Safety, tolerability, pharmacokinetics, and pharmacodynamics, as well as the impact of food on these factors of GS-9674 in healthy volunteers
	NCT02854605	Phase II: Safety, tolerance, and efficacy of GS-9674 in participants with NASH
	NCT04971785	Phase II: Semaglutide, Cilofexor, and Firsocostat fixed-dose combination safety and efficacy study in patients with compensated cirrhosis due to NASH
	NCT02781584	Phase II: Selonsertib, Firsocostat, and Cilofexor in adults with NASH: safety, tolerability, and efficacy
	NCT03987074	Phase II: In participants with NASH, the safety, tolerability, and the efficacy of monotherapy and combination regimens were examined
	NCT03449446	Phase IIb: In people with compensated cirrhosis or bridging fibrosis, the effectiveness and safety of the drugs Selonsertib, Firsocostat, Cilofexor, and combinations for NASH, are being evaluated
EDP-305	NCT03748628	Phase I: The AME (Absorption, Metabolism, and Excretion) study of [14C]EDP-305 in healthy male subjects
	NCT02918929	Phase I: EDP 305 research in subjects with and without presumed NAFLD
	NCT03207425	Phase I: Comparison of participants in the EDP-305 study with mild and moderate hepatic impairment versus healthy people
	NCT03421431	Phase II: Safety, tolerability, pharmacokinetics and effectiveness of EDP-305 in patients with NASH
	NCT04378010	Phase IIb: Safety and effectiveness of EDP-305 in patients with liver-biopsy-confirmed NASH
Px-102	NCT01998672	Phase I: Study using multiple ascending oral doses and Px-102
Px-104	NCT01999101	Phase II: FXR agonist safety pilot study in NAFLD patients
Vonafexor (EYP001)	NCT03976687	Phase I: Examination of the safety, tolerability, pharmacokinetics, and pharmacodynamics of EYP001a in NASH patients and healthy volunteers
	NCT03812029	Phase IIa: EYP001a’s effectiveness, tolerability, and pharmacokinetics in NASH patients
MET409	NCT04702490	Phase IIa: In patients with type 2 diabetes and NASH, MET409 may be used alone or in combination with empagliflozin
TERN-101 (LY2562175)	NCT04328077	Phase IIa: An investigation on the pharmacokinetics, efficacy, and safety of TERN-101 in people with NASH but without cirrhosis

## Data Availability

Not applicable.

## Abbreviations

CYP8B1, sterol 12a-hydroxylase; GCDCA, glycochenodeoxycholic acid; TCDCA, taurochenodeoxycholic acid; BSH, bile salt hydrolase; HSDH, hydroxysteroid dehydrogenase; MCAO, middle cerebral artery occlusion; SAH, subarachnoid hemorrhage; CTX, cerebrotendinous xanthomatosis; TUDCA, tauroursodeoxycholic acid; HD, Huntington’s disease; ALS, amyotrophic lateral sclerosis; HE, hepatic encephalopathy; BDNF, brain-derived neurotrophic factor.
